# Structure-Based Virtual Screening and Functional Validation of Potential Hit Molecules Targeting the SARS-CoV-2 Main Protease

**DOI:** 10.3390/biom12121754

**Published:** 2022-11-25

**Authors:** Balasubramanian Moovarkumudalvan, Anupriya Madhukumar Geethakumari, Ramya Ramadoss, Kabir H. Biswas, Borbala Mifsud

**Affiliations:** 1 Division of Genomics and Translational Biomedicine, College of Health and Life Sciences, Hamad Bin Khalifa University, Qatar Foundation, Education City, Doha P.O. Box 34110, Qatar; 2 Division of Biological and Biomedical Sciences, College of Health and Life Sciences, Hamad Bin Khalifa University, Qatar Foundation, Education City, Doha P.O. Box 34110, Qatar; 3 Biological Sciences, Carnegie Mellon University—Qatar, Qatar Foundation, Education City, Doha P.O. Box 24866, Qatar; 4 William Harvey Research Institute, Queen Mary University of London, Charterhouse Square, London EC1M 6BQ, UK

**Keywords:** COVID-19, SARS-CoV-2 main protease, structure-based virtual screening, molecular docking, FDA-approved drugs, natural products, deep learning, BRET

## Abstract

The recent global health emergency caused by the coronavirus disease 2019 (COVID-19) pandemic has taken a heavy toll, both in terms of lives and economies. Vaccines against the disease have been developed, but the efficiency of vaccination campaigns worldwide has been variable due to challenges regarding production, logistics, distribution and vaccine hesitancy. Furthermore, vaccines are less effective against new variants of the SARS-CoV-2 virus and vaccination-induced immunity fades over time. These challenges and the vaccines’ ineffectiveness for the infected population necessitate improved treatment options, including the inhibition of the SARS-CoV-2 main protease (M^pro^). Drug repurposing to achieve inhibition could provide an immediate solution for disease management. Here, we used structure-based virtual screening (SBVS) to identify natural products (from NP-lib) and FDA-approved drugs (from e-Drug3D-lib and Drugs-lib) which bind to the M^pro^ active site with high-affinity and therefore could be designated as potential inhibitors. We prioritized nine candidate inhibitors (e-Drug3D-lib: Ciclesonide, Losartan and Telmisartan; Drugs-lib: Flezelastine, Hesperidin and Niceverine; NP-lib: three natural products) and predicted their half maximum inhibitory concentration using DeepPurpose, a deep learning tool for drug–target interactions. Finally, we experimentally validated Losartan and two of the natural products as in vitro M^pro^ inhibitors, using a bioluminescence resonance energy transfer (BRET)-based M^pro^ sensor. Our study suggests that existing drugs and natural products could be explored for the treatment of COVID-19.

## 1. Introduction

COVID-19 has caused a devastating effect on global economy and well-being. With 633,750,838 total cases and 6,604,764 total deaths as of 10 November 2022 [1], COVID-19 has led to recession in most countries and strained healthcare systems. The causative agent SARS-CoV-2 is an RNA-virus belonging to the β-coronaviruses with greater infectivity and transmissibility compared to SARS and MERS-CoVs [2]. Early diagnosis of SARS-CoV-2 infection using various diagnostic techniques is crucial to mitigate its community-wide spread [3]. Structural components of coronaviruses include the spike proteins, extracellular membrane, envelope, nucleocapsid and non-structural proteins [4,5]. Infection is initiated by the binding of the spike protein to the angiotensin-converting enzyme 2 (ACE2) receptor on the host cell membrane, followed by internalization of viral RNA. The strategic plan for COVID-19 research by the NIH-NIAID (National Institute of Health-National Institute of Allergy and Infectious Diseases) emphasizes interpretation of the disease characteristics, development of rapid diagnostic kits and effective vaccine development, along with repurposing available drugs to combat COVID-19. A number of vaccines have been developed against the disease.; however, they cannot be used for treatment of the infected segment of the population. While vaccines could protect only the unaffected cohort by initiating immune response against the pathogen, drug repurposing could be an immediate solution for disease management and alleviation in the affected population.

Researchers have investigated the SARS-CoV-2 RNA-dependent RNA polymerase (RdRp), SARS-CoV-2 main protease (M^pro^ or 3CL^pro^) and SARS-CoV-2 receptor binding domain (RBD) as prospective therapeutic targets involved in viral proliferation. The key enzyme SARS-CoV-2 M^pro^ cleaves viral polyproteins translated from the viral mRNA into functional polypeptides required for the assembly of viral progeny [6].

The SARS-CoV-2 M^pro^ is comprised of 306 amino acids encompassing three domains (I-III) with 11 cleavage sites in its substrate, large polyprotein 1ab. Substrate binding is facilitated by the active site harboring the catalytic dyad (His41 and Cys145) in the cleft between domains I and II (Figure S1). In addition, the catalytic activity is induced by dimerization of the enzyme which is prompted by the key residue Glu166 [6]. Targeting the catalytic dyad and the residues in its vicinity and blocking the substrate-binding pocket with an inhibitor lead molecule could render the enzyme inactive.

Thus, the strategy to inhibit the activity of SARS-CoV-2 M^pro^ can greatly mitigate disease progression. Structure-based virtual screening (SBVS) can be used to identify the lead inhibitor molecules against SARS-CoV-2 M^pro^. SBVS for drug discovery involves computational prediction of interactions between a target biological macromolecule serving as the receptor and its lead compounds. Docking algorithms, such as AutoDock [7], calculate the likelihood of a ligand binding to the target macromolecule. High-affinity ligands thus identified could be designated as potential inhibitors of SARS-CoV-2 M^pro^.

Several virtual screening studies attempted to identify potential inhibitors from the available approved drug repositories against SARS-CoV-2 M^pro^. Some of the potential approved drugs which could be repurposed as anti-COVID therapeutics include disomin, hesperidin, MK-3207, dihydroergocristina, bolazine, R228, ditercalinium, etoposide, teniposide, UK-432097, irinotecan, lumacaftor, velpatasvir, eluxadoline, ledipasvir, remdesivir, saquinavir, darunavir, lopinavir, oseltamivir, and ritonavir as predicted by in silico studies [8,9,10]. Additionally, natural products such as flavone and coumarin derivatives [10]; peptides derived from lactoferrin [11] and whey protein [12]; and food components such as sesamin, ellagic acid, epicatechin and capsaicin [13] have also been predicted to have inhibitory activity against SARS-CoV-2 M^pro^. However, most studies did not analyze these predicted inhibitors further.

The current study identifies effective hit inhibitor molecules from the FDA-approved drug library and the natural products library using docking studies with SARS-CoV-2 M^pro^, and predicts these candidate’s effective concentration. Further, the protein–drug interactions were experimentally validated using a BRET-based M^pro^ sensor in vitro.

## 2. Materials and Methods

### 2.1. Retrieval and Preparation of M^pro^ for Virtual Screening

High-resolution X-ray crystal structures of the M^pro^ protein in unliganded (PDB code: 6Y2E at 1.75 Å resolution [6]) and liganded form (PDB code: 6WNP at 1.44 Å resolution) complexed with Boceprevir were retrieved from the Protein Data Bank (PDB) [14]. The extracted PDB coordinates were prepared for docking studies by removal of ligand/heteroatoms/water molecules, and further protonated and Gasteinger charges added using AutoDock tools (Version 1.5.6, Center for Computational Structural Biology, The Scripps Research Institute, La Jolla, CA, USA) [7].

### 2.2. Library Selection

The chemical compound collections of the FDA-approved purchasable drug library (Drugs-lib) and natural products library (NP-lib) inherent in the MTiOpenScreen webserver [15] were chosen, along with the e-Drug3D FDA-approved drug database (https://chemoinfo.ipmc.cnrs.fr/MOLDB/index.php, accessed on 16 August 2020) [16] for the virtual screening.

### 2.3. Ligand Extraction and Preparation

Ligands were extracted from the PubChem database (https://pubchem.ncbi.nlm.nih.gov/, accessed on 15 September 2020) [17] and prepared using AutoDock tools [7].

### 2.4. Docking Parameters

Both 6Y2E and 6WNP were prepared for docking studies as described in Section 2.1. Ligand Boceprevir was downloaded separately from the PubChem database (https://pubchem.ncbi.nlm.nih.gov/, accessed on 3 June 2020) and prepared using AutoDock tools. The prepared Boceprevir ligand was docked with both 6Y2E (free enzyme) and 6WNP (ligand removed) following site direction using grid calculation based on active site/catalytic dyad, and the residues in its vicinity (His 41, Cys 145, His 163, His 164, Met 165, Glu 166) [6] were then compared with the crystal structure 6WNP (M^pro^-Boceprevir complex) for similarity of the interacting amino acid residue environment.

### 2.5. Virtual Drug Screening through Molecular Docking Studies 

Preliminary drug screening was performed using the MTiOpenScreen webservice (https://bioserv.rpbs.univ-paris-diderot.fr/services/MTiOpenScreen/, accessed on 31 August 2020), and the derived optimal drug molecules were further downloaded from the PubChem database to be subjected to molecular docking studies using the MTiAutoDock webservice in-built in the MTiOpenScreen webserver [15].

### 2.6. Analysis and Visualization of M^pro^-Drug Complex 

M^pro^-Drug complexes were analyzed and visualized using PyMoL Molecular Graphics System (Version 2.3, Schrödinger, LLC, New York, NY, USA) and UCSF Chimera (Version 1.15, University of California, San Francisco, CA, USA) [18]. Moreover, the M^pro^-Drug interactions were visualized using Ligplot+ (Version 2.2.4, written by Roman Laskowski, European Molecular Biology Laboratory, European Bioinformatics Institute, Cambridge, UK) [19].

### 2.7. Drug Purchase Information 

FDA-approved drugs Ciclesonide (HY-B0625), Losartan (HY-17512), Hesperidin (HY-15337) and Telmisartan (HY-13955) were obtained from the MedChemExpress LLC (Monmouth Junction, NJ, USA), and the Natural Products (NP1; MolPort-039-052-621, CFN97157) and (NP2; MolPort-039-141-993, CFN97918) were obtained from the MolPort database/ChemFaces Biochemical Co., Ltd. (Wuhan, China).

### 2.8. Prediction of M^pro^-Hit Molecule Interactions Using Deep Learning 

Drug–target interactions between the candidate hit molecule and M^pro^ was predicted using the deep learning tool, DeepPurpose (Version 0.1.5, University of Illinois at Urbana-Champaign, Urbana, IL, USA) [20]. The DeepPurpose tool uses different drug–protein encoder pairs to train five models using its inherent training datasets. The binding affinity of the drug–target pair as one of three binding metrics is predicted using a customizable classifier by applying each of these five models. In this study, the SMILES data of candidate hit molecules and the M^pro^ protein sequence were provided as input to the DeepPurpose tool. The binding affinity of candidate hit molecules and M^pro^ as an IC_50_ metric was predicted by the DeepPurpose tool using five pre-trained DeepPurpose models trained using the BindingDB training dataset. The five pre-trained DeepPurpose models include four drug encoders: the convolutional neural network (CNN), multi-layer perceptrons (MLP) on Morgan, Daylight Fingerprint 1 and the message passing neural network (MPNN), and two protein encoders—CNN and MLP—on amino acid composition (AAC).

### 2.9. Cell Lysate Preparation for In Vitro BRET Assay 

HEK 293T cells were seeded onto 10 cm dishes and transfected with the BRET-based mNG-M^pro^-Nter-auto-NLuc M^pro^ sensor [21] plasmid DNA using 150 μg/dish of the polyethyleneimine (PEI) lipid (Sigma-Aldrich; 408727-100 mL) in Opti-MEM (Invitrogen; 31985088). At 48 h post-transfection, cells were lysed in a buffer containing 50 mM HEPES (pH 7.5), 50 mM NaCl, 0.1% Triton-X 100, 1 mM Dithiothreitol (DTT) and 1 mM ethylenediamine tetraacetic acid (EDTA) [22] on ice after washing with chilled Dulbecco’s phosphate-buffered saline (DBPS). Cell lysates were collected in a 1.5 mL Eppendorf tube and centrifuged at 4 °C for 1 h at 18400× *g*, following which supernatant was collected, aliquoted and stored at −80 °C until further usage.

### 2.10. Expression and Purification of Recombinant SARS-CoV-2 M^pro^

*Escherichia coli* (*E. coli*) BL21-CodonPlus cells (Agilent Technologies) were transformed with the plasmid pETM33_NSP5_M^pro^ (a gift from Ylva Ivarsson (Addgene plasmid #156475)) and the selected colony was inoculated in 100 mL of LB medium. Protein expression was induced by the addition of 0.1 mM isopropyl-β-D-thiogalactopyranoside (IPTG), followed by incubation at 37 °C for 2.3 h. The cells were harvested by centrifugation (4000× *g*, 10 min, 4 °C) and the pellet was resuspended in 10 mL lysis buffer (50 mM Tris (pH 8), 300 mM NaCl, 10 mM beta-mercaptoethanol (bME), 1 mM PMSF and 10% (*v*/*v*) glycerol), followed by sonication. The supernatant was collected after centrifugation (18,000× *g*, 90 min, 45 °C) and incubated with GSH beads at 4 °C for 2 h. The beads were washed (wash buffer—50 mM Tris (pH 7), 150 mM NaCl, 10 mM beta-mercaptoethanol (bME), 1 mM EDTA, 0.01% Triton X-100 and 10% glycerol) and incubated with PreScission Protease (GE Healthcare#27-0843-01) in cleavage buffer (50 mM Tris (pH 7), 150 mM NaCl, 1 mM DTT, 1 mM EDTA, 0.01% Triton X-100 and 10% glycerol) at 4 °C for 16 h. The supernatant containing M^pro^ was collected after centrifugation at 500× *g* at 4 °C for 10 min.

### 2.11. In Vitro BRET-Based M^pro^ Proteolytic Cleavage Inhibitor Assay 

The inhibitors at various concentrations (ranging from 10^−3^ to 10^−10^ M) were prepared from 10 mM stock solutions and incubated with 2 μM of recombinantly purified SARS-CoV-2 M^pro^ protease for 1 h at 37 °C in buffer containing tris-buffered saline (TBS), 1 M sodium citrate, 1 mM EDTA and 2 mM DTT, followed by the addition of cell lysates containing the BRET-based M^pro^ sensor [21]. GC376 (GC376 Sodium; AOBIOUS-AOB36447; stock solution prepared in 50% DMSO at a concentration of 10 mM) at a final concentration of 100 μM was used as a control. BRET measurements were performed at 37 °C by the addition of furimazine (Promega, Madison, WI, USA) at a dilution of 1:200. The bioluminescence (467 nm) and fluorescence (533 nm) readings were recorded using Tecan SPARK multimode microplate reader and used to calculate the BRET ratios (ratio of emission at 533 nm and 467 nm wavelengths). EC_50_ values were calculated using the BRET ratio obtained at 30 min after the addition of the NLuc substrate.

## 3. Results

The SARS-CoV-2 M^pro^ crystal structure 6WNP complexed with Boceprevir was prepared such that the ligand coordinates were removed, retaining only the protein coordinates. Then, the 3D coordinates of Boceprevir were downloaded from the PubChem database. Furthermore, both the protein and ligand were prepared using AutoDock tools as mentioned in Section 2.1 and Section 2.3.

The 6WNP protein was docked with Boceprevir using MTiAutoDock. The docked 6WNP protein–Boceprevir complex was analysed using Ligplot and compared with the original crystal structure of 6WNP (M^pro^–Boceprevir complex). The rationale is to refine the docking parameters such that the docked 6WNP protein–Boceprevir complex was comparable with the crystal structure 6WNP (M^pro^–Boceprevir complex) in terms of the active site interacting residue environment. The same devised parameters have been applied for docking the unliganded SARS-CoV-2 Main Protease crystal structure 6Y2E to screen the potential inhibitors. The unliganded (free enzyme) 6Y2E structural coordinates were taken for further docking studies as the liganded form of 6WNP protein structure’s catalytic site was influenced by the bound ligand Boceprevir.

We chose the purchasable FDA-approved drug library collections e-Drug3D-lib (1993 drugs) and Drugs-lib (7173 drugs), along with natural products database, NP-lib (1228 compounds), as repositories for screening potential inhibitors. To virtually screen lead-like compounds, only the compounds among the above-mentioned databases that comply with physio-chemical properties as previously described [23] were docked with the unliganded (free enzyme) 6Y2E using the refined docking parameters in the MTi-OpenScreen webserver.

From the virtual screening, drug compounds that had a calculated binding affinity >−9 kcal/mol were selected. There were 67 drugs from e-Drug3D-lib, 121 drugs from Drugs-lib and 33 compounds from NP-lib (Table S1). Among these compounds we selected those that are used as anti-virals, respiratory ailments, anti-asthmatics or anti-hypertensives. Further, analogous compounds and those without 3D co-ordinates were also removed from the selected drug list. Thus, we shortlisted 12, 14 and 19 compounds from e-Drug3D-lib, Drugs-lib and NP-lib, respectively (Table S2). Individual 3D structural coordinates for the shortlisted compounds were downloaded from the PubChem database and site-specific docking simulation was performed with 6Y2E using the MTiAutoDock webservice in-built in the MTiOpenScreen webserver.

**Figure 1:**
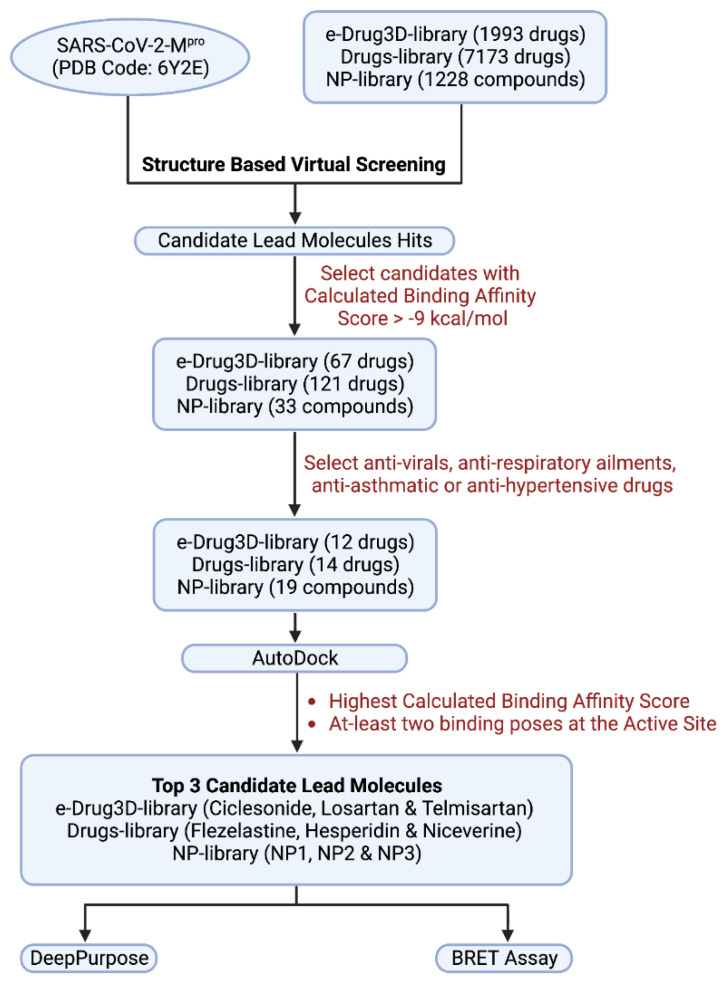
Virtual screening cascade. Flow chart summarizes each stage of the virtual screening steps.

**Figure 2:**
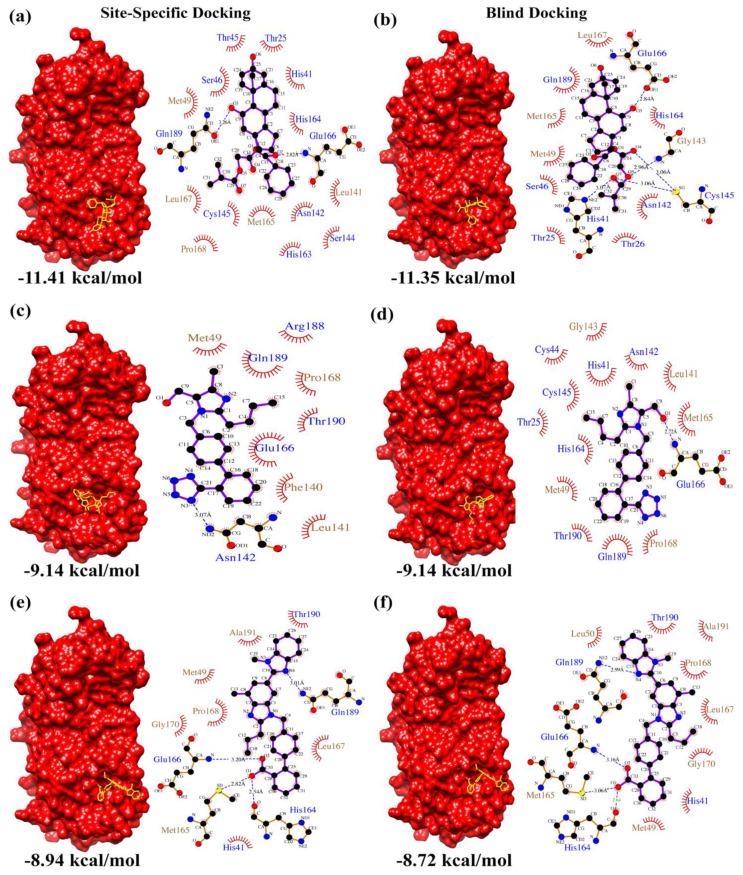
e-Drug3D-lib docking summary. Surface representation of M^pro^ complexed with (**a**,**b**) Ciclesonide, (**c**,**d**) Losartan and (**e**,**f**) Telmisartan resulting from site-specific and blind docking studies, respectively. The corresponding LigPlot interaction maps (polar residue names in blue font and non-polar residue names in brown font) are shown adjacent to the complexes.

**Figure 3:**
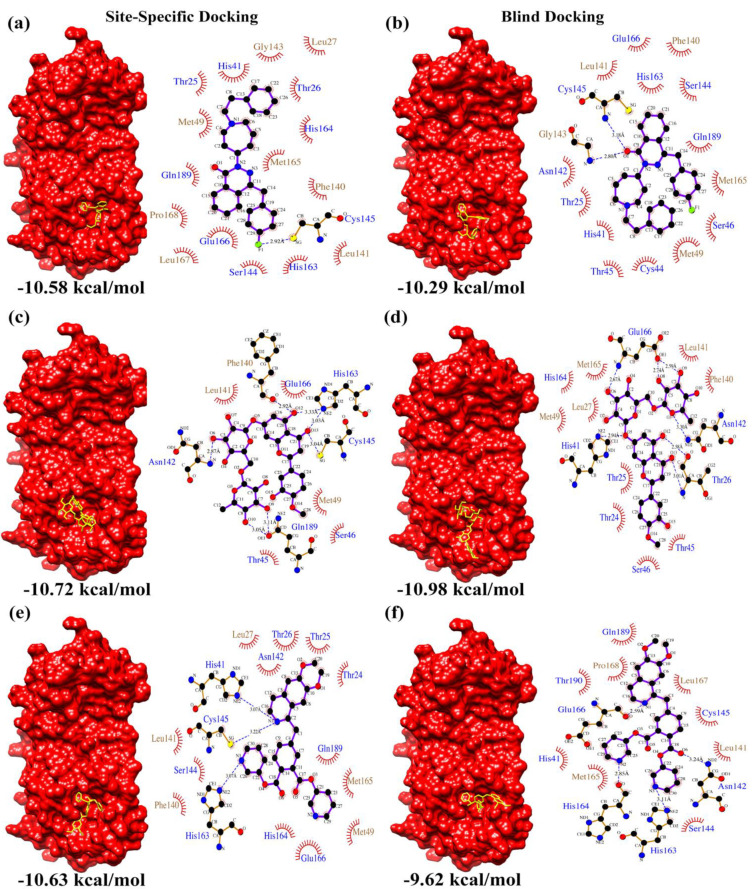
Drugs-lib docking summary. Surface representation of M^pro^ complexed with (**a**,**b**) Flezelastine, (**c**,**d**) Hesperidin and (**e**,**f**) Niceverine resulting from site-specific and blind docking studies, respectively. The corresponding LigPlot interaction maps (polar residue names in blue font and non-polar residue names in brown font) are shown adjacent to the complexes.

**Figure 4:**
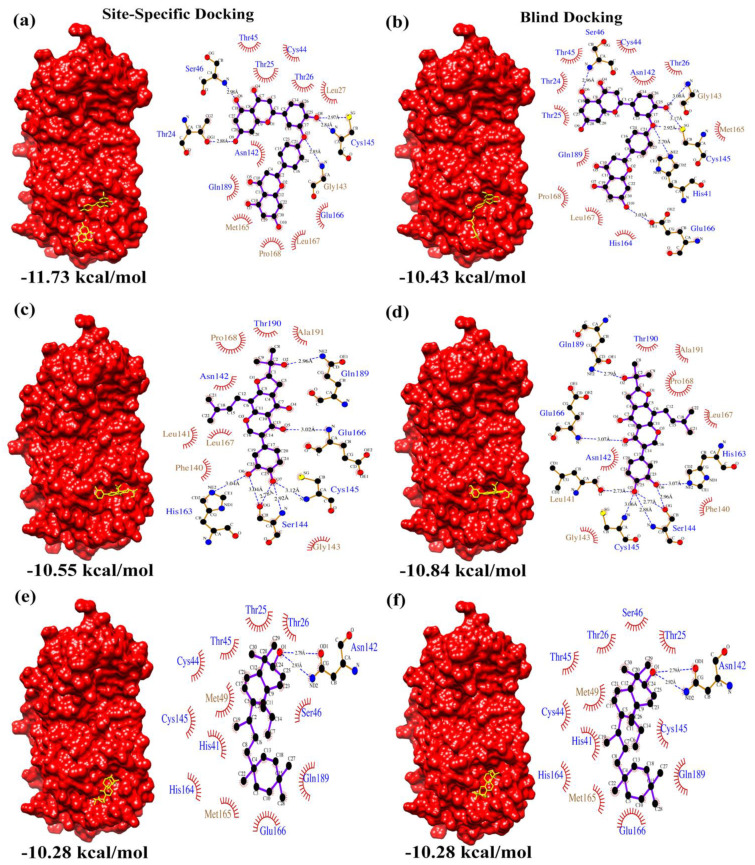
NP-lib docking summary. Surface representation of M^pro^ complexed with (**a**,**b**) NP1, (**c**,**d**) NP2 and (**e**,**f**) NP3 resulting from site-specific and blind docking studies, respectively. The corresponding LigPlot interaction maps (polar residue names in blue font and non-polar residue names in brown font) are shown adjacent to the complexes.

From the docking results, the top three compounds exhibiting the highest calculated binding affinity and at least two poses at the active/catalytic site were chosen [24]. Figure 1 summarizes each stage of the virtual screening cascade. The molecular docking simulation was carried out in triplicates. Additionally, we also conducted blind docking in triplicates to predict the unconstrained protein–drug binding interactions shown in the docking summary in Table 1 and Figure 2, Figure 3 and Figure 4. The shortlisted hit inhibitor molecules were Ciclesonide, Losartan and Telmisartan from e-Drug3D-lib, Flezelastine, Hesperidin and Niceverine from Drugs-lib, and natural products (NP)- natural product compound 1 (NP1), natural product compound 2 (NP2) and natural product compound 3 (NP3) from NP-lib. Further details of the shortlisted drugs are mentioned in Table 2. The selected molecules (NP1, NP2 and NP3) were also studied for their physiochemical and medicinal chemistry properties using the SwissADME server (http://www.swissadme.ch/, accessed on 28 February 2021) [25] shown in the (Figures S2–S4).

In addition to the molecular docking studies, the IC_50_ of shortlisted hit molecules was predicted by using the deep learning tool, DeepPurpose, with the help of five pre-trained models trained using the BindingDB training dataset. BindingDB is a public database containing experimentally determined binding affinities of drug–target interactions with small or drug-like molecules. DeepPurpose predicted median IC_50_ values are listed in Table 3. The predicted median IC_50_ values range between 2–24 μM, with Flezelastine having the lowest predicted IC_50_ value of 2.04 μM and NP1 having the highest predicted IC_50_ value of 23.19 μM. We have also calculated the affinity Kd values from the blind docking scores as represented by Zhang et al. [26].

Furthermore, we validated the inhibitors in vitro using our recently reported BRET-based M^pro^ sensor [21]. BRET is a highly sensitive technique that involves a non-radiative transfer of energy from a donor, a luciferase protein, to an acceptor, a fluorescent protein, depending primarily on their spectral overlap and proximity [27,28,29,30]. It has now been utilized in a number of ways including detection of small molecule ligands [28,30] conformational changes in proteins [29] and monitoring the activity of proteases [31]. In the M^pro^ sensor, the NanoLuc (NLuc) luciferase was used as the energy donor while mNeonGreen (mNG) was used as the energy acceptor and the M^pro^ N-terminal auto-cleavage peptide sequence was included in between the two proteins (Figure S5A). While the intact sensor displays high BRET, proteolytic processing of the cleavage peptide results in a decrease in the BRET. Cell lysate prepared from HEK293T cells expressing the BRET-based M^pro^ sensor and a recombinantly purified M^pro^ protein was used in the assay. BRET measurements were performed after addition of the NLuc substrate. As shown in Figure S5B,C, the BRET ratio of the sensor decreased in the presence of M^pro^, whereas it remained high in the absence of M^pro^. Further, the pharmacological inhibition of SARS-CoV-2 M^pro^ by the known inhibitor-GC376-abrogated the M^pro^-mediated decrease in the BRET ratio (Figures S5B,C).

**Figure 5:**
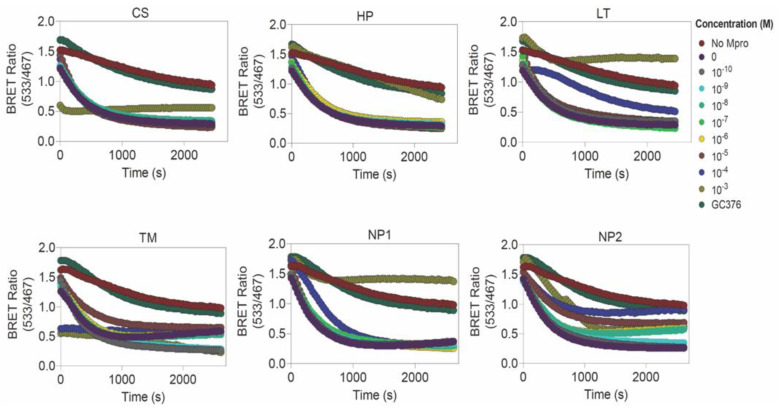
Screening of SARS-CoV-2 M^pro^ inhibitors using a BRET-based sensor. Graph showing time-dependent difference in BRET ratio for various concentrations (10^−3^ to 10^−10^ M) of each inhibitor (CS: Ciclesonide, HP: Hesperidin, LT: Losartan, TM: Telmisartan, NP1: 2,3,2″,3″-Tetrahydroochnaflavone and NP2: Furowanin A) along with GC376 as the control. Graphs also show the decrease in BRET in the presence of M^pro^ and absence of inhibitors.

We then determined the impact of the compounds on SARS-CoV-2 M^pro^ activity by incubating the protease (2 μM) with a range of concentrations of the inhibitors (from 10^−3^ to 10^−10^ M) at 37 °C for 1 h and then monitored time-dependent cleavage of the M^pro^ sensor through BRET (Figure 5). Out of the six compounds, Losartan showed an effective concentration-dependent M^pro^ inhibition with an increase in BRET value (0.69 ± 0.072 at 100 μM and 1.27 ± 0.32 at 1 mM) (Figure 5 and Figure S6) and an approximate 50% reduction in protease activity at 100 μM and 1000 μM (Figure S6), and an EC_50_ value of 260.05 ± 88.60 μM. Additionally, the compound NP1 showed M^pro^ inhibition with an EC_50_ value of 901.1 ± 10.60 μM (Figure 5) with an increase in BRET ratio to 1.22 ± 0.37 (Figure 5 and Figure S6) and a decrease in protease activity to 26% (Figure S6). NP2, on the other hand, showed a lower EC_50_ value (124.8 ± 207.5 μM) and an approximate 50% reduction in M^pro^ activity. Ciclesonide and Hesperidin showed M^pro^ inhibition only at 1 mM concentration and, thus, largely failed to inhibit the protease (Figure 5). Telmisartan completely failed to inhibit M^pro^ (Figure 5).

## 4. Discussion

The main aim of the study is to repurpose FDA-approved drugs, as well as to identify natural product compounds that could inhibit SARS-CoV-2 M^pro^ activity and eventually diminish viral replication. Molecular docking based virtual screening studies help in narrowing down the plausible inhibitory hit molecules against a target from large datasets. The review by Macip et al. [32] have indicated that additional confirmation of docking study results by other computational methods prior to experimental validation is the ideal route in identification of inhibitors against SARS-CoV-2 M^pro^. In this study, we have performed molecular docking studies and prediction of potential inhibitors of SARS-CoV-2 M^pro^ using deep learning followed by experimental validation.

Figure 2, Figure 3 and Figure 4 summarizes the interactions detected in both site-specific and blind docking methods. Most of the lead molecules potentially occupy the active site by interacting with the catalytic dyad and its vicinity residues (His 41, Cys 145, His 163, His 164, Met 165, Glu 166), suggesting strong inhibition of M^pro^ activity.

Among the candidate hit molecules from e-Drug3D-lib, the anti-asthmatic steroid Ciclesonide is presently studied for its therapeutic potential as a nasal inhaler [33]. This was further corroborated by in vitro studies by Matsuyama et al. [34]. Moreover, clinical case studies by Tsuchida et al. [35] and Nakajima et al. [36] support repurposing of Ciclesonide as a potential anti-COVID-19 drug candidate. Meanwhile, Losartan and Telmisartan are angiotensin receptor blockers widely used as anti-hypertensive drugs. Previous studies [37,38] have indicated both drugs to be highly effective in reducing morbidity and mortality in COVID-19 patients. In particular, Losartan has a significant effect on elderly patients [39]. Efficacy of Losartan against SARS-CoV-2 infection was suggested to be ineffective in patients with lung injury [40], but was significant in patients with hypertension [41]. This suggests that the effect of Losartan depends on the pre-existing health condition of the patient, and more clinical studies are required to understand the underlying mechanism.

Among the candidate hit inhibitor molecules from Drugs-lib, Flezelastine is an antihistamine, Hesperidin is an antioxidant/anti-inflammatory agent and Niceverine is an anti-hypertensive. Recent studies suggest that anti-histamine [42] and anti-hypertensive [43] drugs are associated with positive outcomes in COVID-19 patients. Interestingly, the bioflavonoid Hesperidin, prevalent in citrus fruit peels, has been cited to exhibit antiviral properties and is effective in prevention of COVID-19 [44]. Moreover, Hesperidin is also available as a dietary supplement.

Apart from the above-mentioned FDA-approved drugs, natural products were also screened for inhibitory activity against SARS-CoV-2 M^pro^. Among the active natural products screened, Natural Product 1 (NP1) (2,3,2″,3″-Tetrahydroochnaflavone) is an ether-linked bioflavonoid from the *Quintinia acutifolia* tree, endemic to New Zealand [45], and NP2 (Furowanin A) is an isoflavonoid from the leaves of *Millettia taiwaniana Hayata* (Leguminosae) [46].

NP1 scored the highest predicted IC_50_ value (23.19 μM) when assessed using the DeepPurpose deep learning tool, an EC_50_ value of 901.1 ± 10.60 μM was observed from the BRET assay and M^pro^–NP1 docking calculated a binding affinity score of −11.73 kcal/mol (site-specific docking) and −10.43 kcal/mol (blind docking). Moreover, NP2 scored a predicted IC_50_ value (3.45 μM) when assessed using the DeepPurpose deep learning tool, an EC_50_ value of 124.8 ± 207.50 μM was observed from the BRET assay and M^pro^–NP2 docking calculated a binding affinity score of −10.55 kcal/mol (site-specific docking) and −10.84 kcal/mol (blind docking). Thus, these results indicate the potential of NP1 and NP2 to be a plausible inhibitor against SARS-CoV-2 M^pro^. Similarly, Losartan from the e-Drug3D-lib scored a predicted IC_50_ value of 9.1 μM by DeepPurpose, an EC_50_ value of 260.05 ± 88.60 μM was observed from the BRET assay and M^pro^–Losartan docking calculated a binding affinity score of −9.14 kcal/mol in both site-specific and blind docking. Losartan could be repurposed as a potential drug to treat SARS-CoV-2 infection. Further, Losartan, NP1 and NP2 were also checked for complex formation using the EDock program (https://zhanggroup.org/EDock/, accessed on 17 October 2022), which predicts protein–ligand complexes by replica-exchange Monte Carlo simulation [47]. The protein–ligand interacting residue environment was similar to the results obtained from AutoDock, as illustrated in Figure S7. We evaluated the absorption, distribution, metabolism, excretion and toxicity (ADMET) properties and pharmacokinetics of Losartan, NP1 and NP2 using ADMETLab 2.0 (https://admetmesh.scbdd.com/service/evaluation/index, accessed on 19 October 2022) [48] (Table S3). Losartan (Molecular weight: 422.16 g/mol), NP1 (Molecular weight: 542.12 g/mol) and NP2 (Molecular weight: 438.17 g/mol) are accepted as per the Lipinski rule, had an optimal volume distribution, had moderate clearance and are predicted to be respiratory non toxicants.

Only a limited number of studies have utilized BRET assay for screening inhibitors against SARS-CoV-2 M^pro^. Hou, Ningke, et al. [49] have recently evaluated the activity of known SARS-CoV-2 M^pro^ inhibitors such as Boceprevir and GC376 using the BRET assay and have concluded its merit in screening potent inhibitors. The study by Ma, Ling, et al. [50] tested the activity of known HIV/HCV protease inhibitors against SARS-CoV-2 M^pro^. They have deduced the potency of simeprevir against both the SARS-CoV-2 M^pro^ and the mutant Omicron variant M^pro^. Although the SARS-CoV-2 M^pro^ protein of the Omicron variant had a P132H point-mutation, there were no significant structural changes [51], and simeprevir exhibited similar inhibition activity in the BRET assay [50]. The current study, therefore, adds on to the utility of BRET assays for screening inhibitors against M^pro^ [21]. One of the primary advantages of using the BRET-based M^pro^ sensor is that the substrate, which is the sensor, is used at a much lower concentration (~1 nM), likely reflecting the concentration in vivo during SARS-CoV-2 infection compared to the in vitro FRET-based assays (typically 20 or 40 μM). This likely allows detection of impact on M^pro^ activity with both high as well as low affinity lead compounds.

The hits Ciclesonide, Telmisartan, and Hesperidin had good predicted IC_50_ values using the DeepPurpose deep learning tool (Table 3), but no significant activity was observed in the BRET assay despite the good calculated binding affinity scores in docking studies. As shown in the table, Flezelastine scored top as per the predicted IC_50_ values among all drugs, and was followed by Niceverine. Further, the hits Flezelastine, Nicerverine and NP3 scored high predicted binding affinity values by docking studies and predicted IC_50_ values by DeepPurpose, but were not tested in the BRET assay. A notable recent study by Fan and Shi [52] investigated the effect of training datapoints in the efficiency of protein–ligand binding affinity prediction by the DeepPurpose tool. We searched for the human SARS-Corona Virus 3C-like proteinase (3CL^pro^)–ligand datapoints in the BindingDB training dataset used in our study. There were 979 3CL^pro^–ligand pairs present in the dataset, but none of our hit molecules were present (Table S4). Moreover, 149 datapoints were identified with ligands Ciclesonide, Losartan, Telmisartan and Hesperidin interacting with other protein targets. The discrepancy between DeepPurpose and BRET assay results could be due to the lack of datapoints for SARS-CoV-2 M^pro^–hit ligand pairs in the training dataset of the DeepPurpose tool. Experimental validation of the computational predictions is required for better interpretation of inhibitory effects of these drugs on SARS-CoV-2 M^pro^. The computational predictions presented in this study can aid in experimental research activities towards the development of effective SARS-CoV-2 M^pro^ inhibitors.

## 5. Conclusions

Inhibitor molecules against SARS-CoV-2 M^pro^ were identified using structure-based virtual screening of FDA-approved drug libraries (e-Drug3D-lib and Drugs-lib), along with a purchasable natural products library (NP-lib). Among the candidate hit inhibitor molecules, the antihypertensive drug Losartan, the natural product compound 1 (2,3,2″,3″-Tetrahydroochnaflavone; a bioflavonoid) and natural product compound 2 (Furowanin A; an isoflavonoid) exhibited significant inhibitory activity against SARS-CoV-2 M^pro^ as validated by our in-house developed BRET assay. Clinical validation of the efficacy of these compounds against SARS-CoV-2 would be needed to assess their utility as potential drugs.

## Figures and Tables

**Table 1:** SARS-CoV-2 M^pro^–drug docking summary.

**e-Drug3D-lib**	**Ligand**		**Ciclesonide**	**Losartan**	**Telmisartan**
Site-Specific Docking	Calculated Binding Affinity Score (kcal/mol)	1	−11.41	−9.14	−8.94
		2	−11.06	−9.14	−8.29
		3	−11.03	−9.14	−8.02
Blind Docking	Calculated Binding Affinity Score (kcal/mol)	1	−11.35	−9.14	−8.62
		2	−11.09	−9.13	−8.72
		3	−10.88	−9.14	−8.08
**Drugs-lib**	**Ligand**		**Flezelastine**	**Hesperidin**	**Niceverine**
Site-Specific Docking	Calculated Binding Affinity Score (kcal/mol)	1	−10.58	−9.87	−9.60
		2	−10.38	−10.61	−8.90
		3	−10.43	−10.72	−10.63
Blind Docking	Calculated Binding Affinity Score (kcal/mol)	1	−10.29	−10.98	−9.62
		2	−10.10	−9.78	−9.05
		3	−10.00	−9.16	−9.42
**NP-lib**	**Ligand**		**NP1**	**NP2**	**NP3**
Site-Specific Docking	Calculated Binding Affinity Score (kcal/mol)	1	−11.73	−10.55	−10.28
		2	−10.66	−10.37	−10.28
		3	−10.12	−10.47	−10.28
Blind Docking	Calculated Binding Affinity Score (kcal/mol)	1	−10.43	−10.70	−10.28
		2	−10.14	−10.84	−10.28
		3	−10.34	−10.65	−10.28

**Table 2:** Drug information.

Drug Library	Drug/Ligand Name	Drug ID	Clinical Application/Use
e-Drug3D-lib	Ciclesonide	CAS 126544-47-6	Anti-Asthma
	Losartan	CAS 114798-26-4	Anti-Hypertensive
	Telmisartan	CAS 144701-48-4	Anti-Hypertensive
Drugs-lib	Flezelastine	CAS 135381-77-0	Anti-Asthma/Anti-Allergic
	Hesperidin	CAS 520-26-3	Antioxidant/Anti-Inflammatory
	Niceverine	CAS 2545-24-6	Anti-Hypertensive
NP-lib	NP1 (2,3,2″,3″-Tetrahydroochnaflavone)	CAS 678138-59-5 MolPort-039-052-621	-
	NP2 (Furowanin A)	CAS 911004-72-3 MolPort-039-141-993	-
	NP3 (3S,6bS,8aR,12aR,12bS,14bS)4,4,6b,8a,11,11,12b,14b-octamethyl 1,2,3,4,4a,5,6,6b,7,8,8a,9,10,11,12,12a,12b,13,14,14b-icosahydropicen-3-ol	MolPort-002-527-314	-

**Table 3:** Summary of BRET-based SARS-CoV-2 M^pro^ inhibition assay and DeepPurpose results.

Library		Drugs	Determined EC_50_ (μM)	Predicted IC_50_ (μM)	Blind Docking Score (kcal/mol)	Calculated Affinity (Kd) from Blind Docking Score (μM)
e-Drug3D-lib	1	Ciclesonide (CS)	Not determined	6.4	−11.35	0.00469
	2	Losartan (LT)	260.05 ± 88.60	9.11	−9.14	0.196
	3	Telmisartan (TM)	Not determined	3.67	−8.72	0.399
Drugs-lib	1	Flezelastine	Not tested	2.04	−10.29	0.0281
	2	Hesperidin (HP)	Not determined	8.37	−10.98	0.00876
	3	Niceverine	Not tested	3.4	−9.62	0.0872
NP-lib	1	NP1	901.1 ± 10.60	23.19	−10.43	0.0222
	2	NP2	124.8 ± 207.5	3.45	−10.84	0.0111
	3	NP3	Not tested	7.73	−10.28	0.0286

## Data Availability

Not applicable.

## Supplementary Materials

The following supporting information can be downloaded at: https://www.mdpi.com/article/10.3390/biom12121754/s1, Table S1: Virtual screening results data, Table S2: Shortlisted drug compounds after initial virtual screening, Table S3: ADMET results, Table S4: Data points from the BindingDB database, Figure S1: SARS-CoV-2 M^pro^ domain architecture, Figure S2–S4: ADME analysis of NP1, NP2 and NP3; Figure S5: Schematic diagram of BRET-based M^pro^ sensor; Figure S6: Screening of SARS-CoV-2 M^pro^ inhibitors using BRET-based sensor; Figure S7: Comparison of protein–ligand docked complexes.
