# Supplementary material for: Structure-Based Virtual Screening and Functional Validation of Potential Hit Molecules Targeting the SARS-CoV-2 Main Protease
Source: Biomolecules. 2022 Nov 25;12(12):1754. doi: 10.3390/biom12121754 (PMC9775371; doi:10.3390/biom12121754)
Supplement: Supplementary file 1 [file biomolecules-12-01754-s001.zip › Supplement_information.pdf]

Structure-Based Virtual Screening and Functional Validation of Potential Hit Molecules Targeting the SARS-CoV-2 Main Protease

Balasubramanian Moovarkumudalvan[1*], Anupriya Madhukumar Geethakumari[2], Ramya Ramadoss[3], Kabir H Biswas[2] and Borbala Mifsud[1,4*]

[1]Division of Genomics and Translational Biomedicine
[2]Division of Biological and Biomedical Sciences
College of Health and Life Sciences, Hamad Bin Khalifa University
Qatar Foundation, Education City, PO Box 34110. Doha, Qatar.

[3]Biological Sciences, Carnegie Mellon University - Qatar
Qatar Foundation, Education City, PO Box 24866. Doha, Qatar.

[4]William Harvey Research Institute, Queen Mary University of London, Charterhouse Square, EC1M 6BQ, London, UK

*Corresponding Authors: bmoovarkumudalvan@gmail.com, bmifsud@hbku.edu.qa

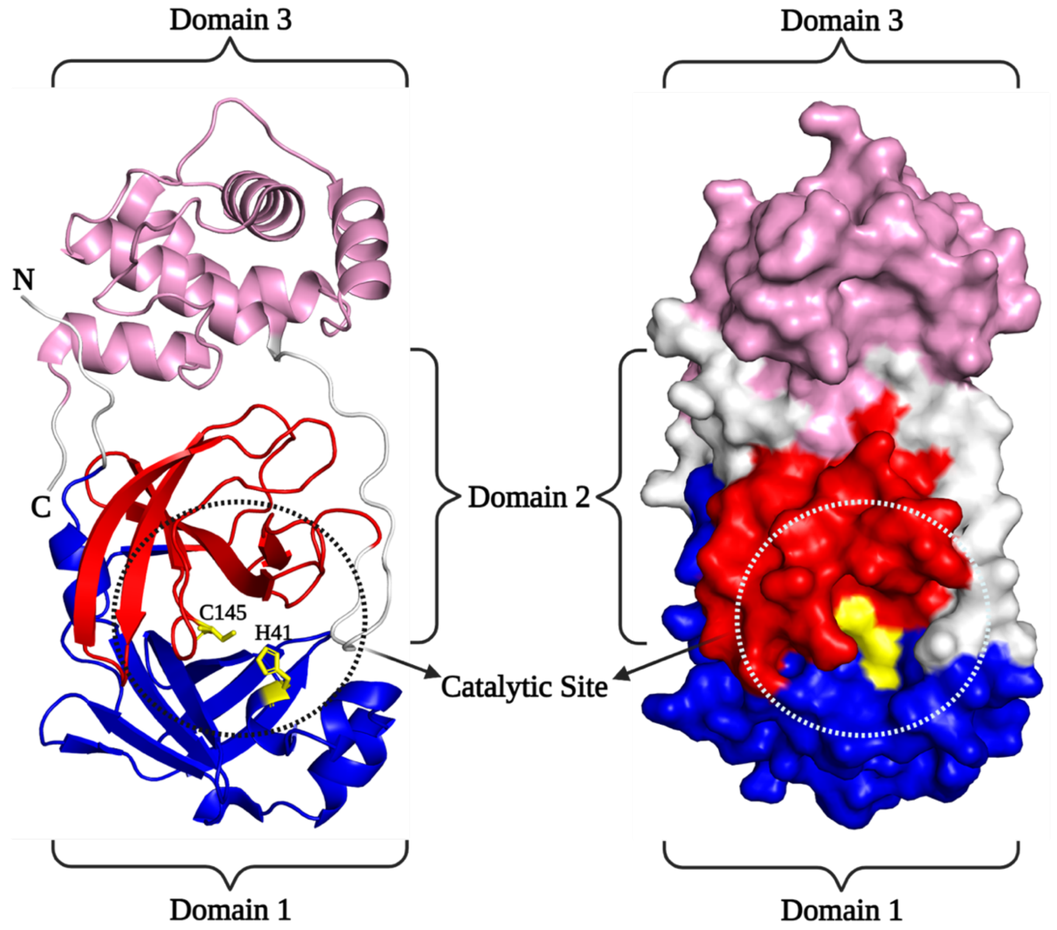

Figure S1. Ribbon (Left) and surface view (Right) of crystal structure of free enzyme SARS-CoV-2 Main Protease (M^{pro}) (PDB code: 6Y2E) with three distinct domains (Domains I, II, and III). Domains I (blue) and II (red), comprised of residues 8–101 and 102–184, respectively, are made up of six antiparallel β-barrels. An antiparallel globular cluster of five α helices forms domain III (pink, residues 201–303) which is connected to domain II *via* a long loop region (cyan, residues 185–200). Active site (highlight by dotted circle) is located in between domains I & II and the His41-Cys145 catalytic dyad is represented in yellow.

S.No	e-Drug3D-library		Drugs-library		NP-library	
	**Drug Name**	**Calculated Binding Affinity Score (kcal/mol)**	**Drug Name**	**Calculated Binding Affinity Score (kcal/mol)**	**Drug Name**	**Calculated Binding Affinity Score (kcal/mol)**
1	Paritaprevir	-11.6	Ecopladib ZINC000085536974	-9.8	MolPort-001-742-110 ZINC000043552589	-10.3
2	Olmesartan	-10.4	Flezelastine ZINC000003785445	-9.7	MolPort-039-052-621 ZINC000044404209	-10.1
3	Voxilaprevir	-10	Velpatasvir ZINC000203686879	-9.3	MolPort-035-705-955 ZINC000003594862	-9.6
4	Tecovirimat	-9.8	Piperaquine ZINC000026251015	-9.3	MolPort-005-909-605 ZINC000036368151	-9.4
5	Glecaprevir	-9.6	Lomitapide ZINC000027990463	-9.3	MolPort-020-005-943 ZINC000031350059	-9.4
6	Ledipasvir	-9.3	Ar-12 ZINC000003960083	-9.3	MolPort-000-741-311 ZINC000253502228	-9.3
7	Losartan	-9.2	Niceverine ZINC000001481885	-9.3	MolPort-001-740-100 ZINC000000603253	-9.2
8	Grazoprevir	-9.1	Elbasvir ZINC000150588351	-9.1	MolPort-039-141-993 ZINC000015205931	-9.2
9	Pibrentasvir	-9.1	Vidupiprant ZINC000043206238	-9.1	MolPort-002-507-280 ZINC000012890906	-9.1
10	Velpatasvir	-9.1	Astemizole ZINC000000601274	-9	MolPort-044-183-452 ZINC000101104515	-9.1
11	Ciclesonide	-9	Grazoprevir ZINC000095551509	-9	MolPort-027-835-585 ZINC000004027386	-9.1
12	Telmisartan	-9	Hesperidin ZINC000008214774	-9	MolPort-035-706-482 ZINC000014766825	-9.1
13			Ledipasvir ZINC000150338819	-9	MolPort-002-526-280 ZINC000253498755	-9
14			Ravidasvir ZINC000150607150	-9	MolPort-001-741-064 ZINC000067902988	-9
15					MolPort-039-141-993 ZINC000015205928	-9
16					MolPort-001-740-946 ZINC000004654620	-9
17					MolPort-002-527-314 ZINC000004082225	-9
18					MolPort-001-768-161 ZINC000001690209	-9
19					MolPort-035-706-502 ZINC000049872145	-9

Table S2. Short listed drug compounds after initial virtual screening.

NP1

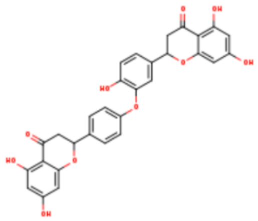

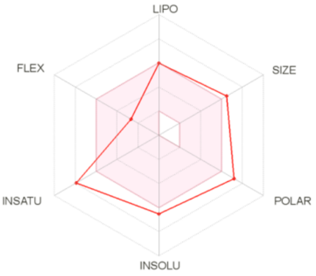

SMILES Oc1cc2OC(CC(=O)c2c(c1)O)c1ccc(cc1)Oc1cc(ccc1O)C1CC(=O)c2c(O1)cc(cc2O)O

Physicochemical Properties	
Formula	C30H22O10
Molecular weight	542.49 g/mol
Num. heavy atoms	40
Num. arom. heavy atoms	24
Fraction Csp3	0.13
Num. rotatable bonds	4
Num. H-bond acceptors	10
Num. H-bond donors	5
Molar Refractivity	141.19
TPSA	162.98 Å²

Lipophilicity	
Log $P_{o/w}$ (iLOGP)	2.62
Log $P_{o/w}$ (XLOGP3)	4.98
Log $P_{o/w}$ (WLOGP)	4.77
Log $P_{o/w}$ (MLOGP)	0.85
Log $P_{o/w}$ (SILICOS-IT)	3.70
Consensus Log $P_{o/w}$	3.38

Water Solubility	
Log *S* (ESOL)	-6.52
Solubility	1.64e-04 mg/ml ; 3.01e-07 mol/l
Class	Poorly soluble
Log *S* (Ali)	-8.14
Solubility	3.92e-06 mg/ml ; 7.22e-09 mol/l
Class	Poorly soluble
Log *S* (SILICOS-IT)	-7.04
Solubility	4.91e-05 mg/ml ; 9.06e-08 mol/l
Class	Poorly soluble

Pharmacokinetics	
GI absorption	Low
BBB permeant	No
P-gp substrate	No
CYP1A2 inhibitor	No
CYP2C19 inhibitor	No
CYP2C9 inhibitor	Yes
CYP2D6 inhibitor	No
CYP3A4 inhibitor	Yes
Log K_p (skin permeation)	-6.07 cm/s

Druglikeness	
Lipinski	Yes; 1 violation: MW>500
Ghose	No; 2 violations: MW>480, MR>130
Veber	No; 1 violation: TPSA>140
Egan	No; 1 violation: TPSA>131.6
Muegge	No; 1 violation: TPSA>150
Bioavailability Score	0.55

Medicinal Chemistry	
PAINS	0 alert
Brenk	0 alert
Leadlikeness	No; 2 violations: MW>350, XLOGP3>3.5
Synthetic accessibility	4.57

Figure S2. Absorption, Distribution, Metabolism, Elimination (ADME) analysis of NP1 using SwissADME webserver.

NP2

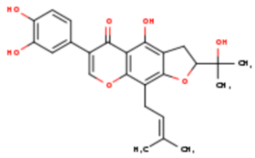

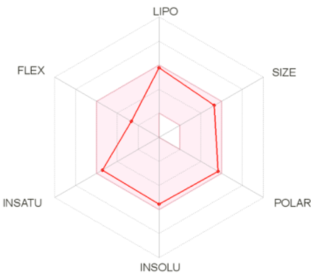

SMILES CC(=CCc1c2OC(Cc2c(c2c1occ(c2=O)c1ccc(c(c1)O)O)O)C(O)(C)C)C

Physicochemical Properties	
Formula	C25H26O7
Molecular weight	438.47 g/mol
Num. heavy atoms	32
Num. arom. heavy atoms	16
Fraction Csp3	0.32
Num. rotatable bonds	4
Num. H-bond acceptors	7
Num. H-bond donors	4
Molar Refractivity	122.67
TPSA	120.36 Å²

Lipophilicity	
Log $P_{o/w}$ (iLOGP)	3.12
Log $P_{o/w}$ (XLOGP3)	4.60
Log $P_{o/w}$ (WLOGP)	4.16
Log $P_{o/w}$ (MLOGP)	1.37
Log $P_{o/w}$ (SILICOS-IT)	4.71
Consensus Log $P_{o/w}$	3.59

Water Solubility	
Log *S* (ESOL)	-5.56
Solubility	1.20e-03 mg/ml ; 2.74e-06 mol/l
Class	Moderately soluble
Log *S* (Ali)	-6.85
Solubility	6.16e-05 mg/ml ; 1.41e-07 mol/l
Class	Poorly soluble
Log *S* (SILICOS-IT)	-5.87
Solubility	5.93e-04 mg/ml ; 1.35e-06 mol/l
Class	Moderately soluble

Pharmacokinetics	
GI absorption	High
BBB permeant	No
P-gp substrate	No
CYP1A2 inhibitor	No
CYP2C19 inhibitor	No
CYP2C9 inhibitor	Yes
CYP2D6 inhibitor	No
CYP3A4 inhibitor	No
Log K_p (skin permeation)	-5.71 cm/s

Druglikeness	
Lipinski	Yes; 0 violation
Ghose	Yes
Veber	Yes
Egan	Yes
Muegge	Yes
Bioavailability Score	0.55

Medicinal Chemistry	
PAINS	1 alert: catechol_A
Brenk	2 alerts: catechol, isolated_alkene
Leadlikeness	No; 2 violations: MW>350, XLOGP3>3.5
Synthetic accessibility	4.63

Figure S3. Absorption, Distribution, Metabolism, Elimination (ADME) analysis of NP2 using SwissADME webserver.

NP3

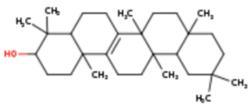

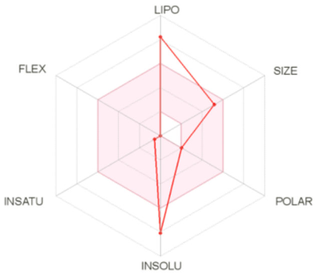

SMILES OC1CCC2(C(C1(C)C)CCC1=C2CCC2(C1(C)CCC1(C2CC(C)(C)CC1)C)C)C

Physicochemical Properties	
Formula	C30H50O
Molecular weight	426.72 g/mol
Num. heavy atoms	31
Num. arom. heavy atoms	0
Fraction Csp3	0.93
Num. rotatable bonds	0
Num. H-bond acceptors	1
Num. H-bond donors	1
Molar Refractivity	134.88
TPSA	20.23 Å²

Lipophilicity	
Log $P_{o/w}$ (iLOGP)	4.71
Log $P_{o/w}$ (XLOGP3)	8.86
Log $P_{o/w}$ (WLOGP)	8.31
Log $P_{o/w}$ (MLOGP)	6.92
Log $P_{o/w}$ (SILICOS-IT)	7.31
Consensus Log $P_{o/w}$	7.22

Water Solubility	
Log *S* (ESOL)	-8.07
Solubility	3.65e-06 mg/ml ; 8.56e-09 mol/l
Class	Poorly soluble
Log *S* (Ali)	-9.17
Solubility	2.88e-07 mg/ml ; 6.76e-10 mol/l
Class	Poorly soluble
Log *S* (SILICOS-IT)	-7.61
Solubility	1.04e-05 mg/ml ; 2.43e-08 mol/l
Class	Poorly soluble

Pharmacokinetics	
GI absorption	Low
BBB permeant	No
P-gp substrate	No
CYP1A2 inhibitor	No
CYP2C19 inhibitor	No
CYP2C9 inhibitor	No
CYP2D6 inhibitor	No
CYP3A4 inhibitor	No
Log K_p (skin permeation)	-2.61 cm/s

Druglikeness	
Lipinski	Yes; 1 violation: MLOGP>4.15
Ghose	No; 3 violations: WLOGP>5.6, MR>130, #atoms>70
Veber	Yes
Egan	No; 1 violation: WLOGP>5.88
Muegge	No; 2 violations: XLOGP3>5, Heteroatoms<2
Bioavailability Score	0.55

Medicinal Chemistry	
PAINS	0 alert
Brenk	1 alert: isolated_alkene
Leadlikeness	No; 2 violations: MW>350, XLOGP3>3.5
Synthetic accessibility	6.04

Figure S4. Absorption, Distribution, Metabolism, Elimination (ADME) analysis of NP3 using SwissADME webserver.

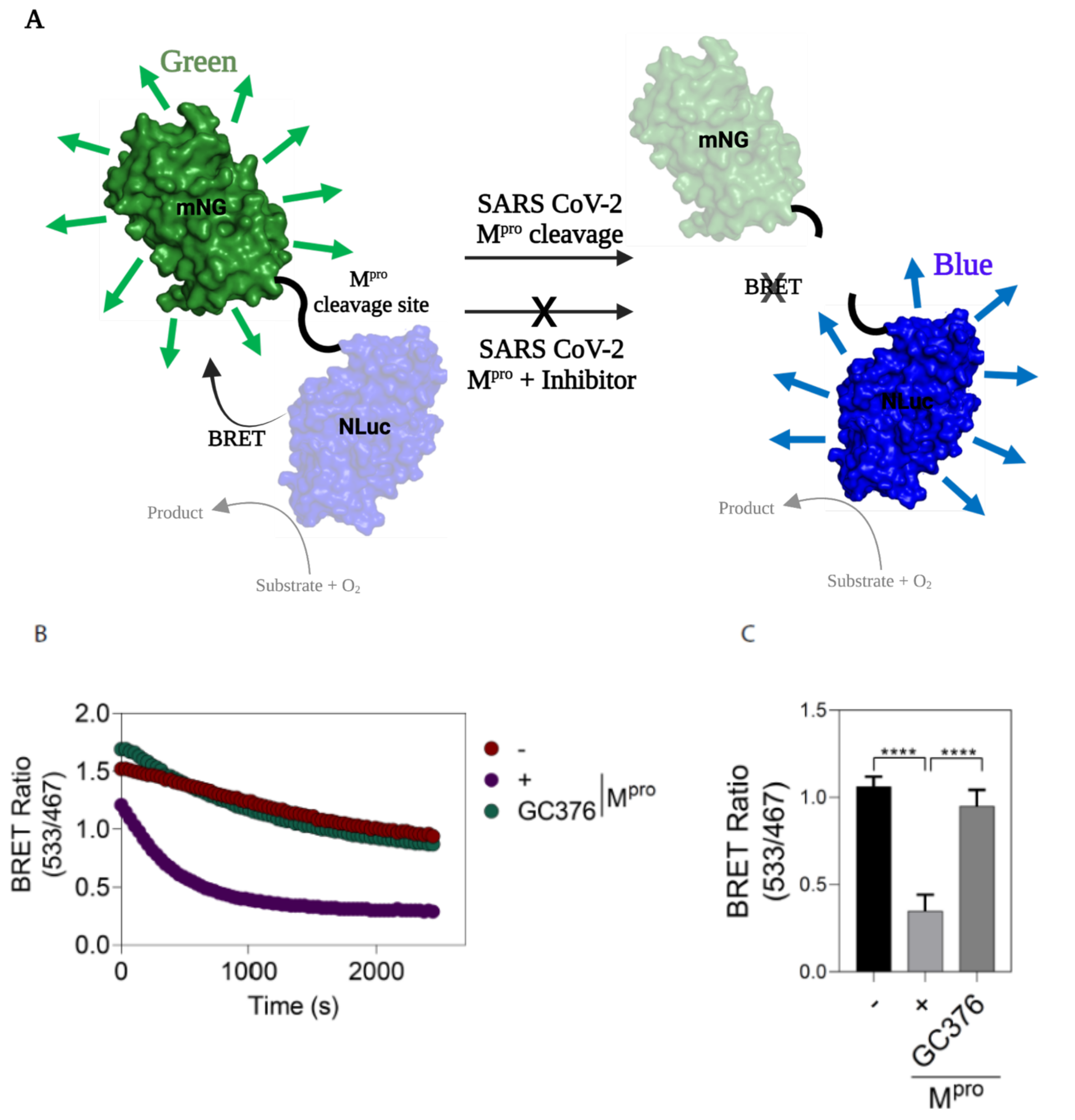

Figure S5. BRET-based M^{pro} sensor for screening the pharmacological inhibition of SARS-CoV-2 M^{pro}

(A). Schematic showing the M^{pro} sensor function. The active M^{pro} protease cleaves the peptide between the NanoLuciferase (NLuc) and mNeonGreen (mNG) causing a decrease in the BRET ratio whereas in presence of M^{pro} inhibitor, the cleavage does not occur resulting in high BRET ratio.

(B). Graph showing the temporal difference in BRET ratio either in presence (purple) or absence (red) of M^{pro} protease or in the presence of SARS-CoV-2 M^{pro} inhibitor – GC376 (green).

(C). Graph showing the inhibition of M^{pro} protease in presence of GC376 after 90 minutes of incubation.

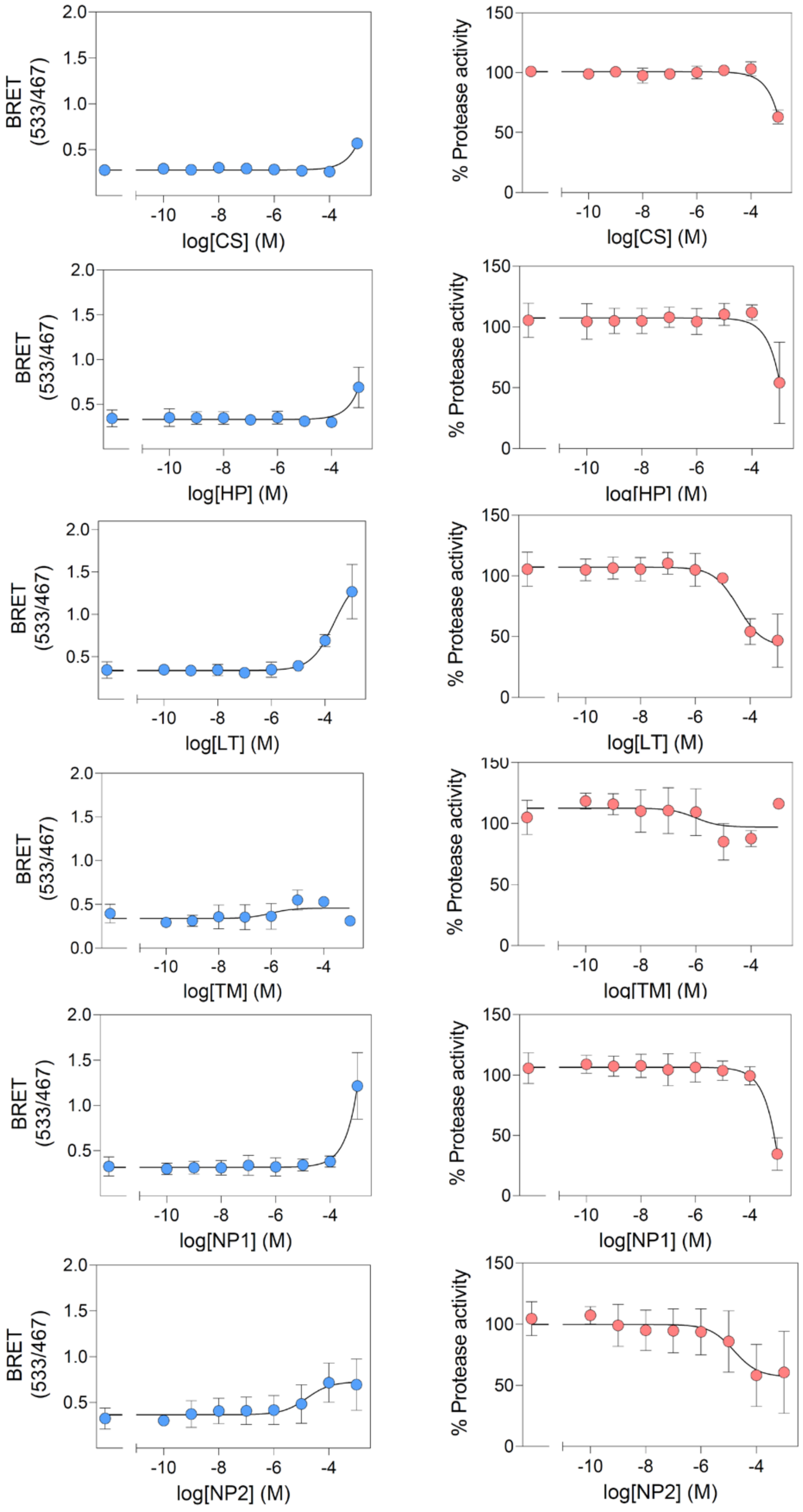

Figure S6. Screening of SARS-CoV-2 M^{pro} inhibitors using BRET-based sensor. Graphs showing BRET ratio (Left side) for each inhibitor concentration at 30 minutes of BRET substrate addition and the difference in percentage of protease activity (Right side) in presence of each concentration of inhibitor.

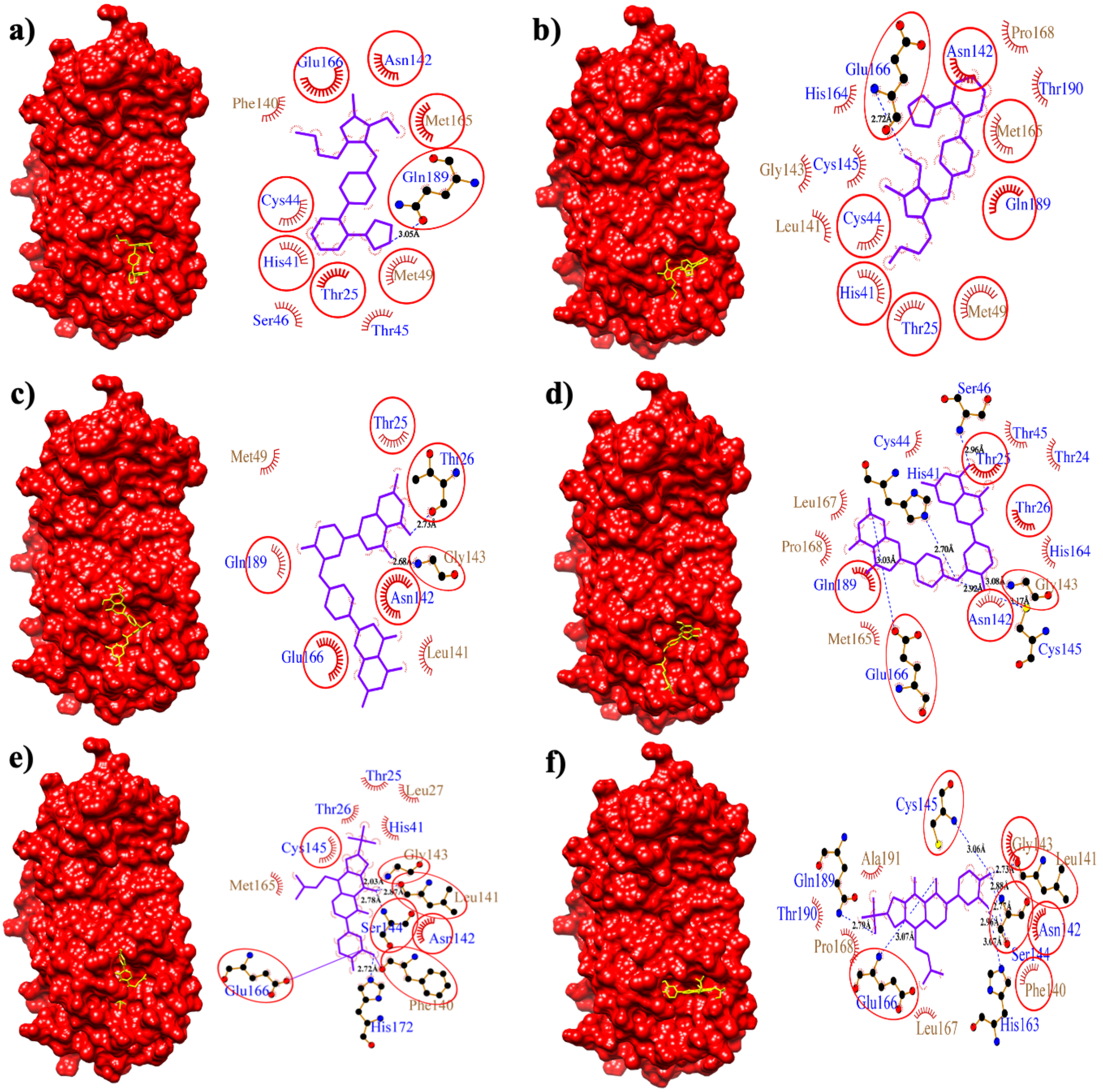

Figure S7. Comparison of protein-ligand docked complexes. Surface representation of SARS-CoV-2 M^{pro} complexed with (a) Losartan, (c) NP1 and (e) NP2 derived from EDock. SARS-CoV-2 M^{pro} complexed with (b) Losartan, (d) NP1 and (f) NP2 derived from AutoDock. Both docking simulations were done in blind docking mode. The corresponding LigPlot interaction maps (polar residue names in blue font & non-polar residue names in brown font) are shown adjacent to the complexes. Residues common among the interacting environments derived from both docking simulations are encircled in red.
